# Case report: Central retinal artery occlusion following scleral buckling surgery with secondary angle closure and recurrent acute ocular hypertension

**DOI:** 10.3389/fmed.2026.1889588

**Published:** 2026-07-02

**Authors:** Jing Chen, Ling Zhang

**Affiliations:** Department of Ophthalmology, People's Hospital of Leshan, Leshan, Sichuan, China

**Keywords:** case report, central retinal artery occlusion, intraocular pressure elevation, post-operative complication, scleral buckling surgery

## Abstract

Central retinal artery occlusion (CRAO) is a rare but vision-threatening complication following retinal detachment surgery. We report a man in his late 60s who underwent combined phacoemulsification with intraocular lens implantation and segmental scleral buckling for macula-sparing rhegmatogenous retinal detachment caused by inferotemporal ora dialysis. Shortly after surgery, he developed recurrent ocular pain with marked intraocular pressure (IOP) spikes, anterior chamber shallowing, iris bombe, and secondary angle closure. Approximately 3 h post-operatively, visual acuity deteriorated to no light perception. Fundus examination showed diffuse retinal whitening, a cherry-red spot, and marked retinal arterial attenuation. Fluorescein angiography demonstrated delayed retinal arterial filling, with focal choroidal hypoperfusion, consistent with CRAO with associated focal choroidal hypoperfusion rather than primary ophthalmic artery occlusion. Anterior segment optical coherence tomography showed forward displacement of the intraocular lens–iris diaphragm and angle closure. The patient was treated promptly with repeated anterior chamber paracentesis, topical pilocarpine, topical and systemic IOP-lowering therapy, intravenous mannitol, oxygen therapy, and adjunctive supportive treatment. IOP gradually normalized, and anterior chamber depth recovered. However, visual recovery was limited, with hand motion vision persisting during follow-up. Although the mechanism cannot be proven definitively, the temporal sequence and anterior segment findings suggest that buckle-related anterior segment crowding may have led to forward displacement of the intraocular lens–iris diaphragm, secondary angle closure, and recurrent acute ocular hypertension, thereby contributing to critically reduced ocular perfusion pressure and retinal ischemia. This case highlights the need for urgent reassessment of IOP, anterior chamber configuration, and retinal perfusion when recurrent ocular pain and shallow anterior chamber occur after scleral buckling surgery.

## Introduction

Rhegmatogenous retinal detachment (RRD) occurs when a full-thickness retinal break allows liquefied vitreous to enter the subretinal space, leading to separation of the neurosensory retina from the retinal pigment epithelium. Surgical management aims to close the retinal break, relieve vitreoretinal traction, and restore retinal attachment (1). Scleral buckling remains an important treatment option for selected cases, particularly when the causative break is peripheral and well localized (2). By creating an external indentation of the sclera, the buckle supports the retinal break and reduces vitreoretinal traction without entering the vitreous cavity. Although scleral buckling is generally effective and safe, it can alter ocular anatomy and influence intraocular pressure (IOP), anterior segment configuration, and ocular perfusion (3). Buckle height, position, and suture tension may contribute to anterior chamber shallowing, secondary angle closure, post-operative ocular hypertension, or impaired ocular circulation in susceptible eyes. Central retinal artery occlusion (CRAO) after scleral buckling is rare but may result in severe and irreversible visual loss. Proposed mechanisms include acute IOP elevation with reduced ocular perfusion pressure, mechanical vascular compromise, perioperative vascular instability, and systemic or hematologic risk factors (4). We report a case of CRAO occurring shortly after combined phacoemulsification with intraocular lens implantation and segmental scleral buckling for localized RRD caused by inferotemporal retinal dialysis. The post-operative course was characterized by recurrent ocular pain, anterior chamber shallowing, iris bombe, secondary angle closure, and recurrent marked IOP spikes before the onset of retinal ischemia. This case suggests a possible pressure-mediated pathway linking post-operative anterior segment crowding to retinal arterial hypoperfusion and highlights the need for early recognition of recurrent ocular pain, shallow anterior chamber, and unstable IOP after scleral buckling surgery.

## Case presentation

### Medical records

**Figure 1:**
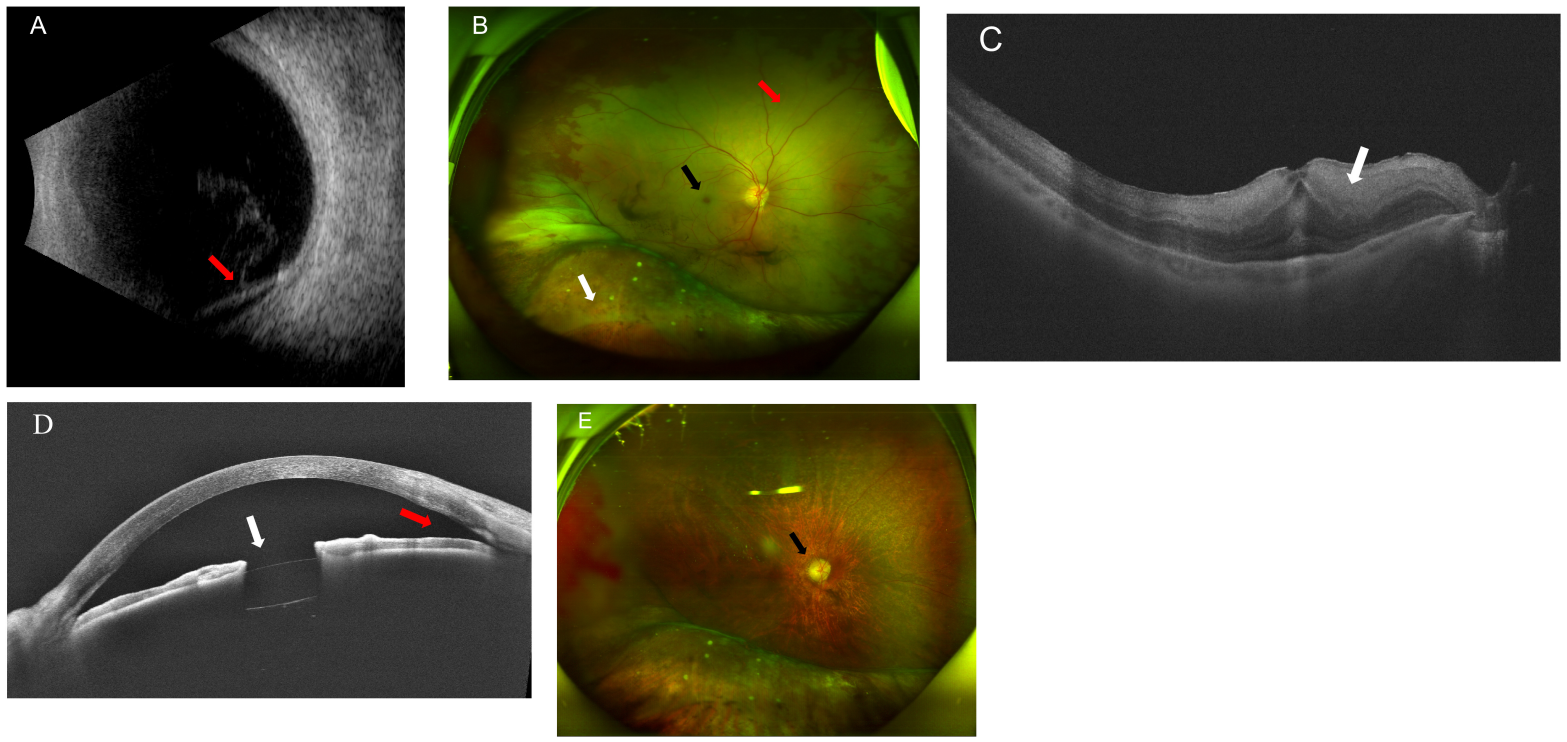
Pre-operative and post-operative imaging findings of the right eye. **(A)** Pre-operative B-scan ultrasonography demonstrating flocculent vitreous opacities and localized peripheral retinal detachment in the inferotemporal quadrant (red arrow). **(B)** Post-operative ultra-widefield fundus photograph demonstrating diffuse retinal whitening (red arrow), a cherry-red spot at the fovea (black arrow), and a prominent inferotemporal buckle ridge. The ora dialysis is positioned on the buckle crest (white arrow). **(C)** Post-operative macular optical coherence tomography demonstrating increased retinal thickness, loss of normal laminar architecture, and diffusely increased inner retinal reflectivity (white arrow) consistent with acute ischemic edema. **(D)** Post-operative anterior segment optical coherence tomography demonstrating forward displacement of the intraocular lens-iris diaphragm (white arrow) and closure of the anterior chamber angle (red arrow). **(E)** At the 2-month follow-up, fundus photography showed resolved retinal edema, attenuated retinal arteries, retinal pigmentary changes, and optic disc pallor (black arrow), consistent with ischemic optic atrophy after central retinal artery occlusion.

The reporting of this case conforms to the CARE guidelines (5). Written informed consent was obtained from the patient for publication of this case report and any accompanying images. All patient details were de-identified. A man in his late 60s presented to the People's Hospital of Leshan, Sichuan, China, in late 2025 with painless visual decline in the right eye for 6 months. He had a history of blunt trauma to the right eye caused by an elbow strike 1 year earlier. He did not notice any decrease in vision after the injury, and the ocular discomfort resolved spontaneously after rest; therefore, no medical consultation was sought at that time. He denied high myopia, hypertension, diabetes mellitus, hyperlipidemia, cardiovascular disease, or cerebrovascular disease. On admission, best-corrected visual acuity was 20/200 in the right eye and 20/32 in the left eye. Intraocular pressure (IOP) was 16 mmHg in the right eye and 17 mmHg in the left eye. Slit-lamp examination of both eyes was unremarkable except for visually significant cataract in the right eye and mild lens opacity in the left eye. The anterior chambers were of normal depth, and pupillary light reflexes were preserved bilaterally. Pre-operative gonioscopy showed a wide-open angle without angle recession. Ultrasound biomicroscopy showed no crystalline lens subluxation or zonular instability. Fundus examination of the left eye was normal, with an attached retina. Pre-operative fundus photography and macular OCT were attempted, but the images were markedly degraded by cataract-related media opacity and were not reliable for documentation or interpretation. Therefore, B-scan ultrasonography was performed and revealed vitreous opacities with a shallow peripheral retinal detachment in the inferotemporal quadrant (Figure 1A). After pharmacologic dilation, three-mirror contact lens examination further demonstrated an attached posterior pole and a retinal dialysis located at approximately the 7-o' clock ora serrata, with localized shallow retinal detachment surrounding the lesion. No macular involvement was observed. Based on these findings, the patient was diagnosed with macula-sparing rhegmatogenous retinal detachment caused by inferotemporal ora dialysis in the right eye, along with visually significant cataract in the right eye and mild lens opacity in the left eye. After comprehensive pre-operative ophthalmic and systemic evaluation revealed no contraindications, combined phacoemulsification with intraocular lens implantation and segmental scleral buckling was planned for the right eye.

### Surgical procedure

Surgery was performed under local anesthesia using a 5-mL mixture of 2% lidocaine and 0.75% ropivacaine administered via retrobulbar injection. Light perception was confirmed after anesthesia. Standard phacoemulsification with posterior chamber intraocular lens implantation (AMO ICB00, +21.50 D) was performed through a clear corneal incision. The intraocular lens was implanted in the capsular bag, and residual ophthalmic viscosurgical device was thoroughly removed. Segmental scleral buckling was subsequently carried out. After conjunctival peritomy and isolation of the inferior and lateral rectus muscles, the inferotemporal retinal dialysis was localized under indirect ophthalmoscopy. Transscleral cryotherapy was applied to the retinal break until a gray-white reaction was observed. A silicone sponge explant measuring approximately 7 × 15 mm was placed from the 5 to 9-o'clock positions, parallel to the limbus. The anterior and posterior edges of the sponge were positioned approximately 7 and 16 mm posterior to the limbus, respectively. The sponge was secured to the sclera with non-absorbable mattress sutures, achieving satisfactory buckle height and indentation. Immediately after conjunctival closure, the patient reported severe ocular pain, accompanied by anterior chamber shallowing and elevated intraocular pressure by palpation. Anterior chamber decompression was promptly performed, resulting in pain relief and normalization of intraocular pressure. Before leaving the operating room, hand motion vision was confirmed, and indirect ophthalmoscopy showed an attached retina, adequate buckle indentation, and well-perfused retinal vessels.

### Post-operative acute event and additional examinations

**Figure 2:**
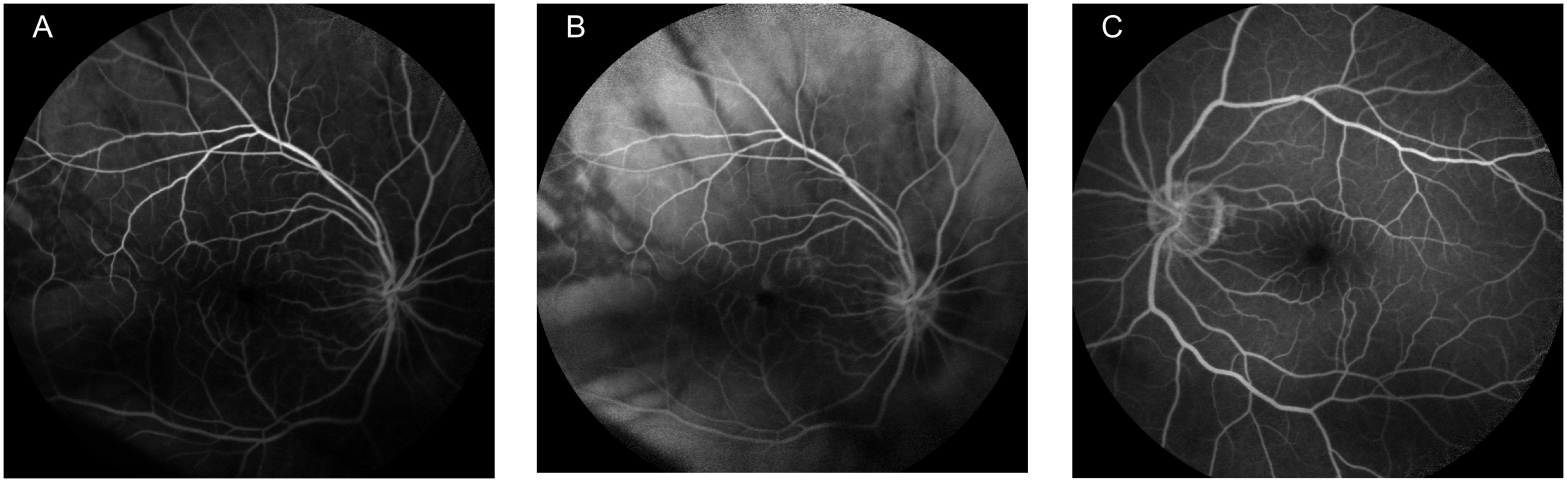
Post-operative fluorescein angiography findings using the Heidelberg Spectralis HRA system. **(A)** Right eye fluorescein angiography showing markedly delayed retinal arterial filling with incomplete arterial perfusion at 28 s post-injection—significantly beyond the normal arterial filling time of 10–15 s. **(B)** Late-phase imaging showed diffuse retinal hyperfluorescence, consistent with retinal ischemia and blood-retinal barrier disruption. Patchy choroidal hypofluorescence was also observed, suggesting concomitant focal choroidal hypoperfusion or ischemia. **(C)** Left-eye fluorescein angiography showing normal retinal arterial filling and perfusion.

The perioperative clinical course, including serial visual acuity, intraocular pressure changes, anterior segment findings, fundus and imaging findings, and acute management, is summarized in Table 1. Time zero was defined as the time when the patient returned to the ward after surgery. During the first 3 h post-operatively, the patient experienced recurrent ocular pain and distension in the right eye, accompanied by repeated elevations in intraocular pressure (IOP) ranging from 30 to 42 mmHg, as measured by tonometry. Each episode was managed with prompt anterior chamber paracentesis, resulting in temporary normalization of IOP. Intravenous 20% mannitol (250 mL) was administered. After each decompression, fundus examination showed a pink retina with no obvious arterial attenuation. Approximately 3 h after surgery, the patient suddenly reported complete vision loss in the right eye. Visual acuity deteriorated to no light perception, and IOP increased to 48 mmHg. Slit-lamp examination revealed diffuse corneal edema, a markedly shallow anterior chamber, iris bombe, and a dilated pupil, with a well-centered intraocular lens. Fundus examination demonstrated diffuse retinal whitening, a cherry-red spot at the macula, and marked attenuation of the retinal arteries, highly suggestive of central retinal artery occlusion (CRAO). Between 5 and 9 h post-operatively, visual acuity partially recovered to light perception, and IOP decreased to 20–28 mmHg with treatment. However, retinal whitening and arterial narrowing persisted. To evaluate potential systemic or embolic causes, echocardiography, carotid Doppler ultrasonography, chest computed tomography, and cranial computed tomography were performed, none of which identified a definite embolic source or major vascular abnormality. Ultra-widefield fundus photography confirmed diffuse retinal whitening with a cherry-red spot and demonstrated a prominent inferotemporal buckle ridge with closure of the original retinal dialysis (Figure 1B). Macular optical coherence tomography showed increased inner retinal reflectivity and thickening consistent with acute retinal ischemia (Figure 1C). Anterior segment optical coherence tomography (AS-OCT) demonstrated forward displacement of the intraocular lens–iris diaphragm and closure of the anterior chamber angle (Figure 1D). Fluorescein angiography revealed markedly delayed retinal arterial filling, with incomplete arterial perfusion at approximately 28 s after dye injection (Figure 2A). Late-phase imaging demonstrated diffuse retinal hyperfluorescence. Patchy choroidal hypofluorescence was also observed (Figure 2B). No abnormalities were observed in the left eye (Figure 2C).

**Table 1:** Clinical timeline of perioperative findings, intraocular pressure changes, imaging examinations, and management.

Time point	Visual acuity	Intraocular pressure	Anterior segment findings	Fundus/imaging findings	Management/clinical notes
Pre-operative examination	20/200	16 mmHg	Clear cornea, normal anterior chamber depth, visually significant cataract	Localized inferotemporal rhegmatogenous retinal detachment caused by ora dialysis; posterior pole and macula attached	Diagnosis of localized retinal detachment with visually significant cataract.
After retrobulbar anesthesia	LP confirmed	Tn by digital palpation	Anterior segment remained stable	Localized inferotemporal retinal detachment	Light perception was confirmed after anesthesia.
After phacoemulsification and IOL implantation	HM confirmed	Tn by digital palpation	Normal anterior chamber depth, IOL well centered in the capsular bag	Localized inferotemporal retinal detachment	Cataract surgery completed before scleral buckling.
End of surgery	HM confirmed	Elevated IOP by digital palpation	Anterior chamber shallowing, ocular pain	Retina attached; retinal vessels appeared well perfused; retinal dialysis located on the buckle crest	Approximately 1 ml of aqueous humor was released through the corneal incision; ocular pain improved and digital IOP returned to Tn.
0–3 h post-operatively	Temporarily preserved vision	30–42 mmHg	Recurrent ocular pain, corneal edema, shallow anterior chamber	After anterior chamber paracentesis, the retina appeared pink and well perfused, and the retinal vessels showed no obvious abnormality	IOP was measured five times during this period, each time ranging from 30 to 42 mmHg. Anterior chamber paracentesis was performed immediately after each elevation, with IOP returning to the normal range after decompression. Intravenous 20% mannitol 250 mL was administered.
Approximately 3 h post-operatively	NLP	48 mmHg	Diffuse corneal edema, shallow anterior chamber, iris bombe, dilated pupil, IOL well centered	Diffuse gray-white retinal edema, cherry-red spot in the macula, and marked narrowing of retinal arteries	CRAO was clinically diagnosed based on sudden severe visual loss, marked IOP elevation, and typical fundus findings. Management was broadened to include intensive IOP-lowering therapy, miotic therapy with topical pilocarpine, oxygen therapy, vasodilator treatment, and neurotrophic support.
5–9 h post-operatively	LP	20–28 mmHg	Mild corneal edema, shallow anterior chamber, dilated pupil, and IOL well centered	Persistent diffuse gray-white retinal edema, cherry-red spot, and narrowed retinal arteries	Systemic and ocular evaluations were completed, including echocardiography, carotid Doppler ultrasonography, chest CT, cranial CT, anterior segment OCT, macular OCT, ultra-widefield fundus photography, and fluorescein angiography. The existing treatment regimen was maintained without modification.
Approximately 9 h post-operatively	LP	18 mmHg	Mild corneal edema, anterior chamber depth improved, and IOL well centered	Retinal ischemic changes persisted	Continued IOP-lowering therapy, miotic therapy, oxygen therapy, vasodilator treatment, and neurotrophic support.
Post-operative day 1	LP	15 mmHg	Clear cornea, restored anterior chamber depth, well-centered IOL, sluggish pupillary light reflex	Retina remained stable	Continued IOP-lowering treatment, miotic therapy, microcirculation-improving therapy, and neurotrophic support.
Post-operative day 3/discharge	HM	13 mmHg	Anterior chamber depth restored, IOL well centered, sluggish pupillary light reflex	Retina remained stable	Continued treatment after discharge, including IOP control, microcirculation support, and neurotrophic therapy. Routine post-operative anti-inflammatory and prophylactic anti-infective eye drops were continued.
2-month follow-up	HM, without further improvement	12 mmHg	Stable anterior segment	Resolution of retinal edema, persistent retinal arterial narrowing, and optic disc pallor	All medications were discontinued. Findings were consistent with optic atrophy after severe ischemic retinal injury.
6-month follow-up	HM, unchanged	Normal range	Stable anterior segment	Fundus findings remained similar to those at 2 months, with persistent retinal arterial attenuation and optic disc pallor	No additional multimodal imaging was performed because visual acuity, IOP, and fundus findings remained stable. No neovascular glaucoma or recurrent angle closure was observed.

Intra-operative hand motion vision was estimated clinically rather than measured using a standard visual acuity chart. It was assessed by confirming light perception and the patient's ability to detect hand movement in front of the operated eye while lying on the operating table.

Time zero was defined as the time when the patient returned to the ward after completion of surgery.

CRAO, central retinal artery occlusion; CT, computed tomography; HM, hand motion; IOL, intraocular lens; IOP, intraocular pressure; LP, light perception; NLP, no light perception; OCT, optical coherence tomography; Tn, normal tension by digital palpation.

### Treatment after CRAO diagnosis and follow-up

When CRAO was clinically diagnosed approximately 3 h after surgery, as indicated by sudden deterioration to no-light-perception visual acuity, diffuse retinal whitening, a cherry-red spot, and marked retinal arterial attenuation, management was broadened from acute IOP control to measures aimed at reducing IOP and improving ocular perfusion. Topical IOP-lowering therapy was initiated in the right eye with timolol, brinzolamide, and brimonidine tartrate twice daily, and pilocarpine nitrate four times daily. Oral acetazolamide was administered twice daily as systemic IOP-lowering therapy. Supplemental oxygen therapy was also provided. As adjunctive vasodilatory treatment, a single 0.5-mg tablet of nitroglycerin was administered sublingually during the acute episode. Compound anisodine injection was then administered by superficial temporal subcutaneous injection on the right side. Oral Ginkgo biloba extract and oral mecobalamin were prescribed as adjunctive treatments to improve microcirculation and provide neurotrophic support, respectively. In addition to the treatments directed at CRAO, the patient received routine post-operative anti-inflammatory and prophylactic anti-infective eye drops for 1 month following combined phacoemulsification and scleral buckling surgery. Approximately 9 h after surgery, IOP decreased to 18 mmHg, and light perception was restored. On post-operative day 1, IOP remained within the normal range, the cornea was clear, anterior chamber depth had returned to normal, and the intraocular lens remained well centered. At discharge on post-operative day 3, visual acuity had improved to hand motion, and IOP was 13 mmHg. Discharge medications included brimonidine tartrate eye drops twice daily in the right eye, oral Ginkgo biloba extract, and oral mecobalamin. Compound anisodine injection was continued in the outpatient setting once daily for an additional 14 days. During follow-up, IOP remained stable without recurrent angle closure. At the 2-month visit, visual acuity in the right eye showed no further improvement. Fundus examination demonstrated resolution of retinal whitening, persistent retinal arterial attenuation, and optic disc pallor, consistent with optic atrophy secondary to severe retinal ischemia (Figure 1E). All medications were discontinued at this visit because IOP remained stable and no recurrent angle closure was observed. At the 6-month follow-up, visual acuity remained hand motion, and IOP was within the normal range. Fundus examination showed no meaningful change compared with the 2-month visit, with persistent retinal arterial attenuation and optic disc pallor. Because visual acuity, IOP, and fundus findings remained stable, no additional multimodal imaging was performed at this visit. No recurrent angle closure, ocular hypertension, or neovascular glaucoma was observed during the follow-up period.

## Discussion

Central retinal artery occlusion (CRAO) is an acute ischemic ocular event and is considered the ocular equivalent of cerebral stroke. Its annual incidence is estimated to be approximately 1–2 cases per 100,000 population, and it most commonly affects older individuals with underlying cardiovascular or cerebrovascular disease (6). CRAO is usually caused by thromboembolic occlusion or, less commonly, vascular spasm, resulting in interruption of retinal arterial blood flow and sudden, painless, usually unilateral visual loss (7). CRAO is an uncommon but devastating complication following retinal detachment surgery (8). Previous reports suggest that arterial occlusion after scleral buckling is rare and often occurs in the presence of additional systemic or ocular risk factors. Aslam et al. (4) reported arterial occlusion after scleral buckling in a patient with retinal dialysis, in whom fluorescein angiography showed delayed filling of both the retinal and choroidal arterial systems. The authors attributed the event to a combination of perioperative hypotension, globe compression, and previously undiagnosed hematologic risk factors, including relative polycythemia and activated protein C resistance. In another recent report, CRAO occurred after scleral buckling combined with expansile gas insertion in a patient with sickle cell disease, and the event was considered related to the combined effects of segmental buckling, gas expansion, relatively low systemic perfusion pressure, and sickle cell vasculopathy (9). In contrast, our patient had no known hematologic disease or sickle cell vasculopathy. The most striking feature in the present case was the immediate post-operative sequence of recurrent ocular pain, shallow anterior chamber, iris bombe, secondary angle closure, and marked IOP elevation before the onset of retinal ischemia.

Ora dialysis is a distinct form of rhegmatogenous retinal detachment and is generally classified as either traumatic or spontaneous. Traumatic ora dialysis usually presents unilaterally and is often localized, with a limited extent of dialysis, supporting the concept that the retinal break is related to a focal point of blunt impact rather than only to intrinsic retinal thinning at the ora serrata. Although ora dialysis may occur in any quadrant, blunt ocular trauma has been reported to more frequently involve the inferotemporal retina, probably because this region is relatively less protected by the orbital rim and may be more exposed during protective upward rotation of the globe. In the present patient, a previous history of blunt trauma to the right eye was identified. However, because he did not notice any visual decline or persistent ocular symptoms at that time and did not undergo fundus examination, a definite causal relationship could not be established. Nevertheless, the unilateral presentation, localized ora dialysis, and inferotemporal location made a traumatic mechanism clinically plausible. In addition, the asymmetric cataract severity between the two eyes may also be consistent with a remote traumatic effect on the crystalline lens. In the present case, the decision to perform combined phacoemulsification with intraocular lens implantation and segmental scleral buckling was clinically justified. Although the retinal detachment was macula-sparing, the pre-operative BCVA of 20/200 was most plausibly explained by the visually significant cataract and cataract-related media opacity rather than macular detachment. The same media opacity also limited fundus photography and pre-operative macular OCT, making these images unreliable for inclusion. Nevertheless, pre-operative B-scan ultrasonography and three-mirror contact lens examination supported an attached posterior pole with localized inferotemporal ora dialysis. Cataract extraction was therefore performed to restore a clear optical medium and improve visual function. At the same time, cataract surgery alone would not have addressed the causative peripheral retinal break and might have allowed progression of the localized detachment. Intraoperative fluctuations in IOP and vitreous movement during cataract surgery could potentially increase vitreoretinal traction, enlarge the peripheral detachment, or allow progression toward the macula, with a risk of irreversible central visual loss (10). For these reasons, segmental scleral buckling was performed during the same session to support the causative retinal break and prevent post-operative progression of the detachment. This approach was considered appropriate because the dialysis was anterior, peripheral, and well localized (11).

The key feature of this case was the early post-operative warning pattern of recurrent ocular pain, progressive anterior chamber shallowing, iris bombe, secondary angle closure, and repeated IOP elevation before the onset of retinal ischemia (12, 13). Because the retinal dialysis was located near the ora serrata, the anterior edge of the silicone sponge was placed approximately 7 mm posterior to the limbus. This relatively anterior buckle position, together with a broad and prominent segmental indentation, may have altered the ocular contour and produced anterior segment crowding (14). In this setting, forward displacement of the intraocular lens–iris diaphragm, post-operative pupillary dilation, and peripheral iris crowding could have directly narrowed or closed the anterior chamber angle, resulting in recurrent acute ocular hypertension (15). The repeated IOP spikes, ranging from 30 to 42 mmHg and peaking at 48 mmHg, could have critically reduced ocular perfusion pressure and precipitated retinal arterial ischemia (6). This pressure-mediated mechanism is strongly supported by the temporal sequence and anterior segment imaging findings, although it cannot be proven definitively from a single case.

Aqueous misdirection, or malignant glaucoma, was an important differential diagnosis because the patient developed acute post-operative IOP elevation, anterior chamber shallowing, and forward displacement of the intraocular lens–iris diaphragm (16). However, several findings made classic malignant glaucoma less likely. First, the patient did not have typical pre-disposing anatomical features. Pre-operative biometry showed an axial length of 24.36 mm, anterior chamber depth of 3.34 mm, and lens thickness of 4.27 mm. Pre-operative gonioscopic assessment showed a wide-open angle, with no evidence of angle recession. These findings did not suggest short axial length, shallow anterior chamber, thick lens, or pre-existing angle-closure configuration. Second, the clinical response was not typical of persistent aqueous misdirection. In malignant glaucoma, miotic agents may worsen anterior chamber shallowing by increasing ciliary block, whereas cycloplegic therapy is usually expected to deepen the anterior chamber (17). In our patient, pilocarpine nitrate was used because the acute angle closure was considered to be mainly related to forward displacement of the intraocular lens–iris diaphragm, pupillary dilation, and peripheral iris crowding rather than aqueous misdirection. After anterior chamber decompression, IOP-lowering therapy, and miotic treatment, the anterior chamber did not become shallower. Instead, IOP gradually decreased, anterior chamber depth recovered, and no recurrent angle closure occurred. This clinical course argued against persistent aqueous misdirection as the dominant mechanism.

Although the patient had a remote history of blunt trauma to the right eye, a lens-related pupillary block was unlikely. Pre-operative ultrasound biomicroscopy showed no evidence of crystalline lens subluxation or zonular instability. In addition, cataract surgery was uneventful, and no intraoperative signs of capsular bag instability or lens displacement were observed. These findings helped exclude lens-induced pupillary block as the major cause of post-operative angle closure. The presence of iris bombe suggested that a transient relative pupillary-block component may have occurred during the acute attack. However, this was most likely secondary to buckle-related anterior segment crowding, post-operative pupillary dilation, and forward displacement of the intraocular lens-iris diaphragm, rather than a primary lens- or intraocular lens-related pupillary block. The rapid recovery of anterior chamber depth and stable IOP without laser peripheral iridotomy further argued against persistent pupillary block as the dominant mechanism. A contribution from ciliary body edema, anterior rotation of the ciliary body, or reduction of the ciliary ring diameter after scleral buckling cannot be fully excluded. These mechanisms have been described as causes of secondary anterior chamber shallowing and elevated IOP after scleral buckling (18). However, the favorable response to pilocarpine and the recovery of anterior chamber depth without the need for cycloplegia, laser peripheral iridotomy, laser capsulotomy, or vitreous decompression did not support clinically significant ciliary block, pupillary block, or persistent aqueous misdirection. Therefore, if ciliary body changes were present, they were more likely to have acted as an additional contributor to anterior segment crowding rather than as the primary mechanism. This interpretation is limited by the absence of post-operative ultrasound biomicroscopy. UBM was not performed because it requires direct contact and pressure on the recently operated eye, which could have increased the risk of wound leakage, corneal incision instability, or intraocular infection. Post-operative gonioscopy was also avoided for the same reason. Therefore, the presence and degree of ciliary body edema, anterior ciliary body rotation, or ciliary sulcus narrowing could not be directly assessed. Non-contact anterior segment optical coherence tomography was used instead and demonstrated forward displacement of the intraocular lens–iris diaphragm and angle closure. Taken together, the anatomical findings and clinical course support buckle-related anterior segment crowding with secondary angle closure as the most likely mechanism of the acute post-operative ocular hypertension in this case.

Other potential mechanisms for post-operative ocular hypertension and CRAO were carefully considered. Retained ophthalmic viscosurgical device is a common cause of early post-operative IOP elevation after cataract surgery. However, in this patient, the OVD was meticulously aspirated from both the anterior chamber and the capsular bag at the conclusion of surgery. Moreover, retained viscoelastic material would not typically account for the marked anterior chamber shallowing, iris bombe, or the degree of anterior segment crowding observed, making this explanation insufficient to fully explain the clinical presentation. Retrobulbar anesthesia has also been associated with retinal arterial occlusion through mechanisms such as acute elevation of orbital pressure, direct vascular compression, vascular spasm, or optic nerve injury (19). In this case, there was no evidence of excessive orbital pressure following anesthesia administration. Light perception was confirmed after anesthesia, hand motion vision was confirmed before leaving the operating room, and retinal perfusion appeared clinically intact at the end of surgery. These findings argued against an immediate anesthesia-related arterial occlusion.

Systemic vascular and embolic causes were also evaluated (20). Pre-operatively, the patient's blood pressure was 124/72 mmHg. Random blood glucose was 7.6 mmol/L, glycated hemoglobin was 5.3%, and the lipid profile was within the normal range. The pre-operative electrocardiogram showed no abnormal findings. Continuous electrocardiographic monitoring during the operation and early post-operative period revealed no arrhythmia, cardiac instability, hypotensive episodes, or other clinically evident cardiogenic abnormalities. Post-operative echocardiography, carotid Doppler ultrasonography, chest CT, and cranial CT did not identify a definite embolic source or major vascular abnormality. These findings made a primary embolic event less likely. However, the systemic evaluation was not exhaustive. Erythrocyte sedimentation rate and C-reactive protein were not measured during the acute episode. Therefore, arteritic CRAO cannot be excluded with complete certainty. Nevertheless, the patient had no systemic symptoms suggestive of giant cell arteritis, such as headache, scalp tenderness, jaw claudication, fever, polymyalgia, or constitutional symptoms. In addition, the immediate post-operative temporal relationship between recurrent marked IOP elevation, secondary angle closure, and retinal ischemia strongly favored an acute pressure-mediated event. Although a multifactorial contribution cannot be completely excluded, the overall clinical course, anterior segment findings, and imaging results collectively support buckle-related anterior segment crowding with secondary acute ocular hypertension as the dominant precipitating mechanism.

The presence of patchy wedge-shaped choroidal hypofluorescence on fluorescein angiography raised the possibility of concomitant choroidal ischemia and prompted consideration of ophthalmic artery occlusion in the differential diagnosis. However, several findings favored CRAO with associated focal choroidal hypoperfusion rather than primary ophthalmic artery occlusion. First, fundus examination demonstrated a typical cherry-red spot at the fovea. This finding suggests preservation of at least part of the choroidal circulation supplying the foveal region and is a classic feature of CRAO. In contrast, ophthalmic artery occlusion usually causes simultaneous interruption of both retinal and choroidal blood flow, often resulting in diffuse retinal whitening without a typical cherry-red spot and absent choroidal perfusion on fluorescein angiography (21). Second, the angiographic abnormalities in our patient were limited to focal areas of choroidal hypoperfusion rather than complete or diffuse choroidal non-perfusion, which would be more characteristic of ophthalmic artery occlusion (22). A pressure-mediated mechanism may also explain the coexistence of retinal and choroidal ischemia in this case. Acute marked elevation of IOP reduces ocular perfusion pressure throughout the eye and may compromise both the central retinal artery and the posterior ciliary circulation. However, the ophthalmic artery is an extraocular vessel located proximal to the intraocular vascular beds. Although markedly elevated IOP can substantially reduce ocular perfusion pressure, it is less likely to directly produce primary ophthalmic artery occlusion. In contrast, elevated IOP may more readily compromise flow within the central retinal artery and posterior ciliary circulation after these vessels enter the globe and become exposed to the altered intraocular pressure environment. The retinal and choroidal circulations also differ substantially in vascular architecture and metabolic characteristics. The retinal circulation is characterized by relatively low blood flow but high oxygen extraction, with an arteriovenous oxygen difference of approximately 40%. In contrast, the choroidal circulation accounts for most ocular blood flow, has exceptionally high flow rates, and extracts only 3%−4% of delivered oxygen (23, 24). Consequently, under conditions of global ocular hypoperfusion, the retina is expected to be more vulnerable to ischemic injury than the choroid. This physiological difference may explain why profound retinal ischemia developed in our patient, whereas the choroidal circulation showed only focal and incomplete hypoperfusion. Taken together, the clinical course, fundus findings, and angiographic features support CRAO secondary to severe pressure-mediated intraocular hypoperfusion, with concomitant focal choroidal ischemia, rather than primary ophthalmic artery obstruction.

Conventional management strategies for CRAO include ocular massage, anterior chamber paracentesis, IOP-lowering therapy, and vasodilatory treatment. However, visual outcomes with these conservative measures are generally poor, particularly when retinal ischemia is severe or prolonged (25). Intra-arterial thrombolysis has been proposed as a treatment option for selected patients with acute CRAO because it may facilitate reperfusion by delivering fibrinolytic agents directly to the ophthalmic circulation (26, 27). Nevertheless, the role of thrombolytic therapy remains controversial, and potential benefits must be weighed against the risk of serious systemic complications. In the present case, the clinical course strongly suggested a pressure-mediated ischemic event secondary to recurrent acute ocular hypertension and critically reduced ocular perfusion pressure rather than an embolic or thrombotic arterial occlusion. Therefore, intra-arterial thrombolysis was not considered the most appropriate treatment strategy. Instead, management was focused on rapid reduction of IOP and restoration of ocular perfusion. The patient received repeated anterior chamber paracentesis, miotic therapy with topical pilocarpine, topical and systemic IOP-lowering therapy, intravenous mannitol, oxygen therapy, and adjunctive vasodilatory and neurotrophic treatment (28, 29). Despite prompt intervention, visual recovery remained limited, which is consistent with the poor prognosis associated with severe retinal ischemia (30).

This case highlights several important clinical considerations. Post-operative ocular pain after scleral buckling should not be regarded as routine discomfort, particularly when it is recurrent or associated with anterior chamber shallowing. IOP, anterior chamber depth, pupil configuration, and retinal arterial perfusion should be assessed repeatedly during the early post-operative period. When recurrent acute ocular hypertension occurs with a shallow anterior chamber after scleral buckling, medical therapy and anterior chamber paracentesis may not be sufficient if the underlying mechanism includes mechanical anterior segment crowding. In such situations, the buckle height, position, and tension should be reassessed promptly, and early buckle adjustment or loosening may need to be considered if IOP cannot be rapidly and stably controlled. Careful pre-operative assessment of anterior segment anatomy, including anterior chamber depth, angle configuration, axial length, and glaucoma risk, may also help identify patients at higher risk for post-operative angle closure after combined cataract and scleral buckling surgery.

## Conclusion

This case reports CRAO occurring shortly after combined phacoemulsification and segmental scleral buckling for localized retinal dialysis. The temporal sequence of recurrent post-operative ocular pain, anterior chamber shallowing, iris bombe, secondary angle closure, marked IOP spikes, and retinal ischemia suggests a pressure-mediated mechanism related to post-operative anterior segment crowding. Although the mechanism cannot be proven definitively, buckle-related forward displacement of the intraocular lens–iris diaphragm appears to be the most plausible precipitating factor. Recurrent ocular pain with a shallow anterior chamber and unstable IOP after scleral buckling should prompt urgent reassessment of IOP, anterior segment configuration, and retinal perfusion. In selected cases, early reconsideration of buckle height, position, or tension may be necessary if IOP cannot be rapidly and stably controlled. Awareness of this potential pathway may help clinicians recognize high-risk post-operative eyes before irreversible retinal ischemic injury occurs.

## Patient perspective

The patient expressed disappointment regarding the severe and permanent visual loss despite prompt treatment. He understood the rarity and seriousness of the complication and agreed to share his clinical course in the hope that it may help improve awareness and management of similar cases in the future.

## Data Availability

The original contributions presented in the study are included in the article/supplementary material, further inquiries can be directed to the corresponding author.
